# Notes and Notices

**Published:** 1883-12

**Authors:** 


### NOTES AND NOTICES.

Errata.—November number, p. 355, line ninth from bottom, for *sepia*$^{2cm}$ read *sepia*$^{c}$. Page 320, line seventh, for *undilutable,* read *undilatable.* Page 318, line twenty-four, for *proven* read *provers.*

J. Marion Sims.—By the death of Dr. Sims, Allopathy loses one of its greatest gynæcologists—a class of men who have done more to debauch, unsex, and demoralize women than any set of men who ever lived. Of these, Dr. Sims was the greatest slasher of all. He had long enjoyed celebrity as a surgeon, skillful and original, and will be especially remembered as the founder of the Woman's Hospital of New York.

A Second Jenner.—Jenner taught physicians to poison mankind with vaccine virus. He gathered his wisdom from the cowboys of Old England. A second Jenner has now appeared in the person of the great Dr. John C. Peters. Dr. Peters advocates the use of an equine (asinine would be better) virus for the purpose of antidoting or preventing scarlatina. He gathers his wisdom from a Russian veterinary surgeon who noticed the outbreak of scarlet fever in various supposably healthy places. He familiarized himself with the habits of life of the first victim in each locality, and states that he was surprised to find that in every case they had been in daily close communication with horses. It also appeared that the animals were at the time or had been previously affected by a disease which was sufficiently like scarlet fever to characterize it as such.

